# Trends and Predictors of Emergency Department Outcomes in Atrial Fibrillation: A Statewide Analysis from North Carolina—2016 to 2023

**DOI:** 10.1016/j.acepjo.2025.100279

**Published:** 2025-11-27

**Authors:** Shantanu Srivatsa, Parsa Pazooki, Benjamin K. Lau, Anna Waller, Anna Bess Brown, Kimberly McDonald, Anil Gehi, Wayne Rosamond

**Affiliations:** 1 UNC Chapel Hill School of Medicine, Gillings School of Public Health, Chapel Hill, North Carolina, USA; 2 UNC Chapel Hill School of Medicine, Chapel Hill, North Carolina, USA; 3 Carolina Center for Health Informatics in the Department of Emergency Medicine, School of Medicine, University of North Carolina at Chapel Hill, Chapel Hill, North Carolina, USA; 4 Division of Public Health, North Carolina Department of Health and Human Services, Raleigh, North Carolina, USA; 5 Division of Cardiology, Department of Medicine, University of North Carolina at Chapel Hill, Chapel Hill, North Carolina, USA; 6 Department of Epidemiology, Gillings School of Global Public Health, University of North Carolina at Chapel Hill, Chapel Hill, North Carolina, USA

**Keywords:** Atrial fibrillation, Heart failure, Epidemiology, Syndromic surveillance

## Abstract

**Objectives:**

The population burden of atrial fibrillation (AF) continues to rise, leading to significant implications for emergency departments (EDs) managing acute presentations and complications. In this study, we aimed to characterize statewide trends in ED visits, admissions, and mortality related to AF, evaluate the association of AF outcomes with heart failure (HF) subtypes, and identify demographic and clinical predictors of these outcomes.

**Methods:**

This retrospective statewide analysis utilized ED data from the North Carolina Disease Event Tracking and Epidemiologic Collection Tool (NC DETECT) from 2016 to 2023. AF encounters were identified using ICD-10-CM diagnosis codes (I48.0-I48.2). Multivariable logistic regression was performed to evaluate predictors of hospital admission and mortality.

**Results:**

Among 39,445,680 ED visits, 517,722 (1.3%) had a diagnosis of AF. AF-related visits peaked in 2018 and declined significantly during the COVID-19 pandemic but recovered to previous levels by 2023. Hospital admission rates remained consistently high (63.7%-73.3%), whereas intra-encounter mortality was low but increased after 2020 (0.2% [pre-2020] to 0.6% [post-2020]). Patients with concomitant HF had increased odds of admission (HfpEF, OR 2.16; 95% CI [2.11-2.21]; HfrEF, OR 2.43; 95% CI [2.36-2.50]). In models evaluating intra-encounter mortality, AF + HFrEF (OR, 1.30; 95% CI [1.05-1.60]) but not AF + HFpEF (OR, 1.13; 95% CI [0.92-1.37]) was significantly associated with increased mortality; in subanalyzes excluding infectious presentations, AF + HFrEF was still associated with increased mortality (OR, 1.56; 95% CI [1.20-2.03]).

**Conclusion:**

These findings highlight continued clinical complexity and disparities in AF management in a large statewide syndromic surveillance database, particularly the association of comorbid HF and AF in emergent settings. Population-wide surveillance of AF can help assess the need for improved outpatient care, especially in populations with comorbid HF or high-risk and underserved populations.

**The Bottom Line**

This retrospective statewide study from North Carolina sought to examine trends in atrial fibrillation (AF) in emergency department settings over the last 7 years. Over the last 7 years, hospitalization rates for patients with AF have remained consistently high. Patients with AF and heart failure, especially those with reduced ejection fraction, had twice the odds of hospital admission and higher mortality risks, highlighting the need for improved management strategies.

## 1 Introduction

### 1.1 Background

The national burden of atrial fibrillation (AF) continues to grow, with recent estimates suggesting over 10.55 million people are currently affected in the United States alone.^1^^,^^2^ As AF becomes more prevalent, its associated complications including stroke, heart failure (HF), and increased mortality may also rise substantially.^3^, ^4^, ^5^

### 1.2 Importance

Given these trends and comorbid conditions, emergency departments (EDs) play a significant role in managing acute or emergent AF presentations. Advances in rate and rhythm control strategies, early initiation of anticoagulation, and improved outpatient care have significantly improved acute AF care.^6^^,^^7^ However, comprehensive population-based surveillance data evaluating how changes in prevalence have affected ED presentations remain limited.

HF specifically is both a cause and result of AF, creating a bidirectional relationship in which each condition promotes the onset and progression of the other.^8^ AF can precipitate or worsen HF through tachycardia-mediated cardiomyopathy and impaired ventricular filling, whereas HF can foster AF development via structural remodeling. This interplay leads to more frequent hospitalizations, greater symptom burden, and higher mortality risk. Although AF and stroke risk have been well-characterized in emergent settings, there are fewer descriptions of AF and comorbid HF, particularly using population-wide syndromic surveillance data.

### 1.3 Goals of This Investigation

To understand trends in statewide AF burden, we conducted an analysis of AF-related ED visits in North Carolina from 2016 to 2023. Our objectives were to characterize NC trends in AF ED visits, admissions, and mortality; characterize its association with HF; and identify demographic and clinical predictors of these outcomes.

## 2 Methods

### 2.1 Study Design and Setting

We conducted a retrospective study using ED encounter data from the North Carolina Disease Event Tracking and Epidemiologic Collection Tool (NC DETECT). NC DETECT is a statewide syndromic surveillance tool that includes ED encounters within the state of NC from all civilian hospital-affiliated EDs (*N* = 131). Staff at the Carolina Center for Health Informatics in the UNC Department of Emergency Medicine (CCHI), under contract to the North Carolina Division of Public Health (NC DPH), develop and manage NC DETECT. In 2005, a statewide mandate, the North Carolina Hospital Emergency Surveillance System (NCHESS), was put in place requiring all civilian EDs in North Carolina to submit select ED data elements to the state for public health surveillance. CCHI monitors the quality of the NCHESS data and works with the North Carolina Healthcare Association, hospitals, and their vendors to ensure NC DETECT users have access to the most accurate data possible. Information collected within the database includes deidentified patient identification number, age, sex, race, county of residence, transportation, arrival time/date, insurance, chief complaint, systolic/diastolic blood pressure (BP), initial ED temperature, disposition, and International Classification of Diseases (ICD-9/10) Diagnosis and Procedural codes. This study was reviewed by the UNC Chapel Hill Institutional Review Board (IRB # 24-2365) and determined to be exempt.

### 2.2 Selection of Participants

Encounters were restricted to patients presenting to a North Carolina ED between January 1, 2016, and December 31, 2023. ED visits for AF were identified using ICD-10-CM diagnosis codes (I48.0-I48.2). Patients with mitral stenosis or mechanical valves were excluded (I05, Z952-954). Concurrent infectious etiology was characterized as ICD-10 codes (A00-B99), and AF with rapid ventricular response (RVR) was determined as I48.91. ED visits for concomitant heart failure were identified as having ICD-10-CM diagnosis codes (I50.x) and classified as heart failure with reduced ejection fraction (HFrEF [I50.2]) or heart failure with preserved ejection fraction (HFpEF [I50.3]). These categories were chosen based on previous literature which have used “systolic” and “diastolic” HF to represent HFrEF and HFpEF, respectively, with reasonable predictive value.^9^

### 2.3 Measures and Outcomes

Covariates included age, biological sex (male or female), race (White, Black, Asian, Native American, and other), insurance type (Private, Medicaid, Medicare, and other), and clinical values (systolic BP, diastolic BP, and initial ED temperature).

Intra-encounter mortality was defined using the standardized discharge disposition field and due to the limitations of the dataset indicated that a patient death occurred during the ED encounter itself and excluded death during subsequent hospital admission or following discharge. Annual mortality was defined as the number of AF ED visits resulting in death in the ED divided by the total number of AF ED visits during that year, expressed as a percentage. Mortality trends were also stratified by sex. Patients with mitral stenosis or mechanical valves were excluded from models examining the risk of admission or mortality.

### 2.4 Data Analysis

Descriptive statistics were used to characterize AF ED visits by calendar year and demographic characteristics. To explore predictors of admissions and mortality, patients with any AF ICD-10-CM code between 2016 and 2023 were included and a multivariable logistic regression model using data from all AF and HF ED presentations was utilized, adjusting for all covariates described previously. Subgroup analyses were conducted excluding AF patients with a copresenting infectious disease diagnosis code. All analyses were conducted using RStudio (version 2024.12.0 + 467), with the *MASS*, *survival*, and *survminer* packages. Visualization was performed using *ggplot2* and *cowplot*. A two-sided *P* value <.05 was considered statistically significant.

## 3 Results

### 3.1 Baseline Characteristics

Between 2016 and 2023, there were a total of 39,445,680 ED visits in North Carolina, of which 517,722 had a diagnosis code for AF, representing 1.3% of all ED encounters. The mean age of patients presenting with AF was 74.8 years (SD 12.2), and approximately 49.8% were male overall. The majority of AF patients were White (range, 80.4%-83.4%), with smaller proportions of Black (15.4%-17.7%), Asian (0.3%-0.4%), and Native American (0.6%-1.7%) patients. Medicare was the predominant insurer among AF patients, accounting for >70% of visits (range, 70.1%-81.1%). There were a total of 56,568 patients with AF with RVR and 81,088 patients with concurrent infectious etiology across the 8-year period. Baseline characteristics, stratified by year, are shown in Table 1.

**Table 1:** Demographic characteristics of ED visits for AF by year.

Characteristic		2016	2017	2018	2019	2020	2021	2022	2023
Total no. of ED visits		5,126,102	5,191,546	5,084,986	5,154,088	4,202,652	4,611,127	4,986,071	5,073,108
No. of ED visits for AF		52,191	67,490	74,355	71,194	46,322	61,653	70,575	73,942
Age (SD)		74.75 (12.38)	74.85 (12.35)	74.73 (12.31)	74.65 (12.24)	73.83 (12.42)	74.68 (12.17)	75.09 (11.97)	75.19 (11.76)
Sex	Male (%)	25,633 (49.1)	32,854 (48.7)	36,801 (49.5)	34,976 (49.1)	23,207 (50.1)	31,175 (50.6)	35,654 (50.5)	37,723 (51.0)
Race	White (%)	43,448 (83.2)	56,144 (83.2)	61,236 (82.4)	58,788 (82.6)	37,248 (80.4)	51,276 (83.2)	58,731 (83.2)	61,695 (83.4)
	Black (%)	8017 (15.4)	10,383 (15.4)	11,632 (15.6)	11,253 (15.8)	8179 (17.7)	9575 (15.5)	10,997 (15.6)	11,527 (15.6)
	Asian (%)	157 (0.3)	265 (0.4)	260 (0.3)	258 (0.4)	171 (0.4)	242 (0.4)	311 (0.4)	312 (0.4)
	Native American (%)	569 (1.1)	698 (1.0)	1227 (1.7)	895 (1.3)	724 (1.6)	560 (0.9)	536 (0.8)	408 (0.6)
	Other (%)	0 (0.0)	0 (0.0)	0 (0.0)	0 (0.0)	0 (0.0)	0 (0.0)	0 (0.0)	0 (0.0)
Insurance	Private (%)	6386 (12.2)	8875 (13.2)	11,522 (15.5)	11,397 (16.0)	7760 (16.8)	10,292 (16.7)	10,212 (14.5)	7897 (10.7)
	Medicaid (%)	1934 (3.7)	2633 (3.9)	2750 (3.7)	2756 (3.9)	2164 (4.7)	2284 (3.7)	2329 (3.3)	2316 (3.1)
	Medicare (%)	36,861 (70.6)	49,321 (73.1)	52,869 (71.1)	49,894 (70.1)	32,983 (71.2)	44,768 (72.6)	54,547 (77.3)	59,979 (81.1)
	Other (%)	7010 (13.4)	6661 (9.9)	7214 (9.7)	7147 (10.0)	3415 (7.4)	4309 (7.0)	3487 (4.9)	3750 (5.1)
Vitals (median, [IQR])	Systolic BP	131.00 (111.00, 151.00)	133.00 (113.00, 151.00)	133.00 (112.00, 152.00)	134.00 (112.00, 154.00)	136.00 (115.00, 156.00)	134.00 (113.00, 153.00)	127.00 (99.00, 150.00)	106.00 (74.00, 140.00)
	Diastolic BP	74.00 (64.00, 87.00)	75.00 (65.00, 87.00)	76.00 (65.00, 89.00)	77.00 (66.00, 89.00)	77.00 (66.00, 88.00)	78.00 (66.00, 92.00)	84.00 (69.00, 108.00)	96.00 (73.00, 121.00)
	Initial ED temp	36.70 (36.50, 36.90)	36.70 (36.50, 37.00)	36.70 (36.50, 37.00)	36.70 (36.50, 37.00)	36.80 (36.60, 37.00)	36.70 (36.50, 36.90)	36.70 (36.50, 36.90)	36.70 (36.50, 36.90)

AF, atrial fibrillation; ED, emergency department.

### 3.2 Trends in ED Visits and AF Admissions and Mortality

The number of ED visits for AF increased from 52,191 visits in 2016 to a peak of 74,355 in 2018, before declining after the COVID-19 pandemic to 46,322 visits in 2020. ED visits for AF rose again to 73,942 visits in 2023, similar to previous highs (Table 2). Admissions remained consistently above 70% across the study period, ranging from 63.7% to 73.3% annually (Table 2).

**Table 2:** Annual admission and mortality proportions of ED presentations for AF in NC (2016-2023)

Year	Total ED AF visits	No. of admissions	No. of deaths	Admission (% of ED AF visits)			Mortality (% of ED AF visits)		
				Overall	Male	Female	Overall	Male	Female
2016	52,191	36,825	120	70.6	71.3	69.8	0.2	0.3	0.2
2017	67,490	43,739	199	64.8	65.2	64.5	0.3	0.3	0.3
2018	74,355	47,495	175	63.9	64.4	63.4	0.2	0.3	0.2
2019	71,194	51,315	46	72.1	72.6	71.6	0.1	0.1	0.1
2020	46,322	33,958	31	73.3	74.0	72.6	0.1	0.1	0.1
2021	61,653	39,272	403	63.7	63.9	63.5	0.7	0.7	0.6
2022	70,575	47,565	396	67.4	68.1	66.6	0.6	0.5	0.6
2023	73,942	51,687	414	69.9	70.0	69.8	0.6	0.6	0.5

AF, atrial fibrillation; ED, emergency department; NC, North Carolina.

Intra-encounter mortality was low throughout the study period but increased after 2020, ranging from 0.1% to 0.7%. The highest annual mortality was observed in 2021 (0.7%), followed by 2022 and 2023 (0.6%) (Table 2).

When stratified by sex, admission rates were slightly higher in males than in females during most years, with both sexes showing similar trends over time, and negligible mortality differences (Table 2).

### 3.3 Predictors of Admission and Mortality

**Figure 1:**
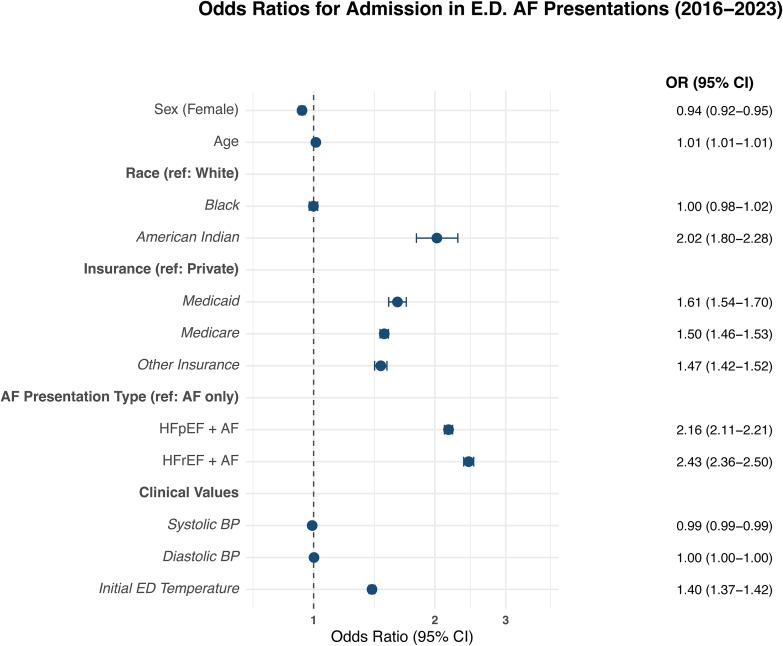
Odds ratios and 95% CIs showing predictors of hospital admission among patients presenting with atrial fibrillation (AF) to North Carolina emergency departments (2018-2023). Higher odds indicate a greater likelihood of hospital admission. Results are adjusted for demographic and clinical covariates. Abbreviations: AF, atrial fibrillation; BP, blood pressure; ED, emergency department; HFpEF, heart failure with preserved ejection fraction; HFrEF, heart failure with reduced ejection fraction; OR, odds ratio.

**Figure 2:**
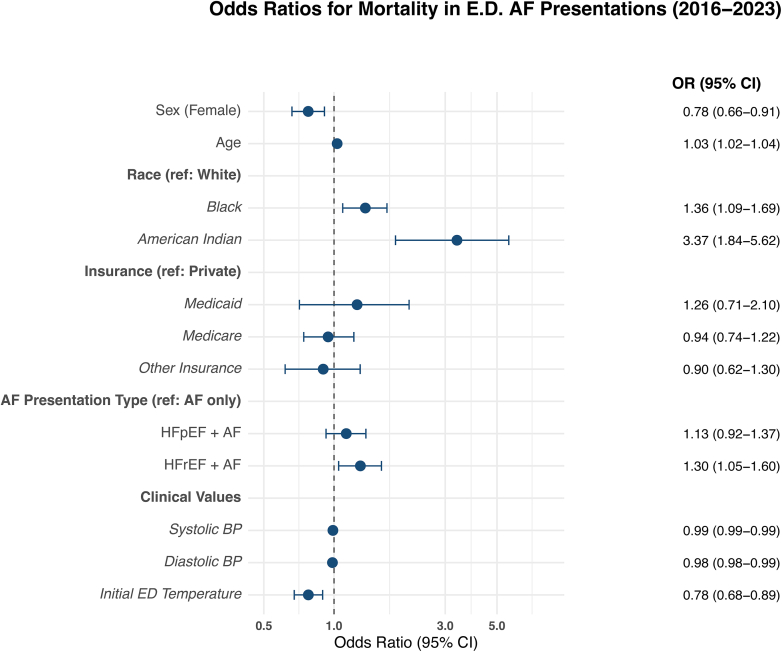
Odds ratios and 95% CIs demonstrating predictors of intra-encounter mortality among patients presenting with atrial fibrillation (AF) to North Carolina emergency departments (2018-2023). Higher odds reflect a greater risk of mortality during the ED encounter. Estimates are adjusted for demographic and clinical covariates. AF, atrial fibrillation; BP, blood pressure; ED, emergency department; HFpEF, heart failure with preserved ejection fraction; HFrEF, heart failure with reduced ejection fraction; OR, odds ratio.

Among AF ED visits between 2016 and 2023, patients who presented with AF and concomitant HF as primary reasons for presentation had greater odds of admission vs AF only (Fig. 1). For those who were subsequently admitted, patients with AF + HFpEF (OR, 2.16; 95% CI [2.11-2.21]) and AF + HFrEF (OR, 2.43; 95% CI [2.36-2.50]) had increased odds of admission compared with patients with AF only. In models evaluating intra-encounter mortality, AF + HFrEF (OR, 1.30; 95% CI [1.05-1.60]) but not AF + HFpEF (OR, 1.13; 95% CI [0.92-1.37]) was significantly associated with increased mortality (Fig. 2).

However, given atrial fibrillation could occur secondarily to infectious causes, we performed subanalyzes examining AF encounters without co-occurring infectious conditions (*N* excluded = 81,088, ICD-10 codes A00-B99). These analyses recapitulated findings in the main analyses; with regard to admission, patients with AF + HFpEF (OR, 2.29; 95% CI [2.23-2.34]) and AF + HFrEF (OR, 2.60; 95% CI [2.53-2.68]) remained having increased odds of admission compared with patients with AF only. As compared with patients with AF only, AF + HFrEF was still significantly associated with increased mortality (OR, 1.56; 95% CI [1.20-2.03]), whereas AF + HFpEF (OR, 1.19; 95% CI, 0.92-1.54) was not.

Among demographic and clinical covariates, age (OR, 1.01; 95% CI, 1.01-1.01), female sex (OR, 0.94; 95% CI, 0.92-0.95), Medicare insurance (OR, 1.50; 95% CI, 1.46-1.53), Medicaid (OR, 1.61; 95% CI, 1.54-1.70), and other insurance (OR, 1.47; 95%C I, 1.42-1.52) vs private insurance, and elevated initial triage temperature (OR, 1.40; 95% CI, 1.37-1.42) were also associated with subsequent hospital admission. Age (OR, 1.03; 95% CI, 1.02-1.04), female sex (OR, 0.78; 95% CI, 0.66-0.91), Black (OR, 1.36; 95% CI, 1.09-1.69) and Native American (OR, 3.37; 95% CI, 1.84-5.62) vs White race/ethnicity, and ED temperature (OR, 0.78; 95% CI, 0.68-0.89) were significant predictors of mortality.

## 4 Limitations

Several limitations should be acknowledged. First, the syndromic surveillance data were restricted to the ED encounter itself and post-discharge mortality could not be evaluated. Because a significant portion of AF diagnosis and presentation is handled in outpatient settings, we were unable to ascertain population incidence rates. In addition, key clinical variables such as heart rate, echocardiographic parameters, anticoagulation use, and comorbid heart failure were unavailable, limiting our ability to capture case complexity and introducing potential misclassification of HF due to incorrect ICD-10-CM coding. In addition, syndromic surveillance data do not allow for identification of a primary diagnosis, and thus, AF could have been either a primary diagnosis or an incidental/contributing condition. Finally, although our data are comprehensive for North Carolina, results may not generalize to other states with differing healthcare access and population characteristics.

## 5 Discussion

We performed a statewide evaluation of over 3 million atrial fibrillation ED visits across an 8-year period to characterize the evolving trends and outcomes of AF in emergency care. Overall, AF visits accounted for 1.3% of all ED visits, approximately 3.5 times larger than a previous national study conducted between 2007 and 2014 in which AF visits accounted for 0.4% of all emergency room encounters.^10^ This statewide rise coincides with other literature, which suggests that the national burden of AF may be growing and could represent ∼5% of the US population.^1^ Although AF presentations increased between 2014 and 2019, the onset of the COVID-19 pandemic coincided with a marked decrease in AF-related ED visits. These findings are consistent with broader national trends of reduced ED utilization for non-COVID conditions during the pandemic timeframe.^11^^,^^12^ Despite the fluctuations in encounter volume, admission rates remained high, suggesting a persistent burden of AF on inpatient services.

The persistently high admission rates following ED encounters exceeding 70% in most years highlight the continued acuity and complexity of AF presentations. We found comparable estimates between female and male intra-encounter admissions and mortality. The transient increases in mortality observed during 2021 and 2022 may reflect delayed presentation during pandemic surges or improved outpatient care, leading to selection bias of patients now represented in emergent setings.

Black and Native American race/ethnicity were among the strongest predictors of both hospital admission and mortality in patients with AF, potentially indicating persistent disparities in cardiovascular care and outcomes for these populations, consistent with national findings.^13^^,^^14^ These disparities are likely multifactorial, driven by systemic inequities in healthcare access, socioeconomic disadvantage, and a higher burden of comorbid conditions. However, sample sizes for these ethnicities were small (<2%) and thus inferences regarding these disparities remain challenging. Collectively, these findings suggest the need for improved access to longitudinal outpatient care and targeted ED interventions for these higher-risk populations.

The bidirectional nature of AF and HF is well known, and the markedly elevated admission risk observed for patients with HFrEF and HFpEF suggests that over time, the presence of both comorbid conditions significantly drives the need for inpatient management. Most notably, AF + HFrEF demonstrated increased mortality risk, particularly in noninfectious presentations, whereas AF + HFpEF was significantly associated with greater admission risk compared with patients with AF only. Some studies have found elevated short-term mortality risk among HFrEF patients compared with HFpEF patients in emergency settings, and this may reflect differing distributions of underlying comorbidities and severity of presentations by HF subtype.^15^^,^^16^

Our study highlights significant trends in the incidence and outcomes of AF in EDs utilizing syndromic surveillance data. AF-related ED visits increased notably prior to the COVID-19 pandemic, subsequently declining but remaining substantial. Patients with concurrent HF, particularly those with reduced ejection fraction, experienced markedly higher hospital admission rates and increased intra-encounter mortality, notably in noninfectious presentations. These findings underscore the continued clinical complexity of AF presentations in ED settings.

## Funding and Support

NC DETECT is a statewide public health syndromic surveillance system, funded by the North Carolina Division of Public Health (NCDPH) Federal Public Health Emergency Preparedness Grant and managed through collaboration between NCDPH and the UNC-Chapel Hill Department of Emergency Medicine’s Carolina Center for Health Informatics. The scientific validity or accuracy of methodology, results, statistical analyses, or conclusions presented are those of the authors and do not necessarily represent the views of the North Carolina Department of Health and Human Services or the Division of Public Health.

## Conflict of Interest

All authors have affirmed they have no conflicts of interest to declare.
